# Clinico-epidemiological profile of patients at children’s psychosocial care centers in São Bernardo do Campo: a cross-sectional study

**DOI:** 10.1590/1516-3180.2021.0577.R1.17022022

**Published:** 2022-08-01

**Authors:** Maialu Pedreira Messias, Etienne de Miranda e Silva, Mariele Hertha de Andrade, Isabela Cristina Casanova Cavallo, Lucas Pequeno Galvão, Cintia de Azevedo Marques Périco, Julio Torales, Antonio Ventriglio, João Mauricio Castaldelli-Maia, Anderson Sousa Martins-da-Silva

**Affiliations:** I MD. Assistant Psychiatrist, Health Secretariat of São Bernardo do Campo, São Bernardo do Campo (SP), Brazil.; II MD. Assistant Psychiatrist, Health Secretariat of São Bernardo do Campo, São Bernardo do Campo (SP), Brazil.; III MD. Assistant Psychiatrist, Health Secretariat of São Bernardo do Campo, São Bernardo do Campo (SP), Brazil.; IV MD. Assitant Physician, Departamento of Psychiatry, Universidade Nove de Julho, São Paulo (SP), Brazil.; V MD. Psychiatry Resident, Health Secretariat of São Bernardo do Campo, São Bernardo do Campo (SP), Brazil.; VI MD. Mental Health Coordinator at the City Hall of Sao Bernardo do Campo, Health Secretariat of São Bernardo do Campo, São Bernardo do Campo (SP), Brazil.; VII PhD. Coordinator of the Department of Psychiatry, Department of Psychiatry, School of Medical Sciences, National University of Asunción, Asunción, Paraguay.; VIII PhD. Assistant Psychiatrist, Department of Clinical and Experimental Medicine, University of Foggia, Foggia.; IX PhD. Postgraduate Professor, Department of Psychiatry, Faculdade de Medicina, Universidade de São Paulo, São Paulo, SP, BR.; X MD. Master’s Student, Department of Psychiatry, Faculdade de Medicina, Universidade de São Paulo, São Paulo, SP, BR.

**Keywords:** Precision medicine, Child, Adolescent, Mental health services, Epidemiology, Health profile, Children, Teen, Mental hygiene services

## Abstract

**BACKGROUDS:**

Child and Adolescent Psychosocial Care Centers (Centros de Atenção Psicossocial, CAPSI) are dedicated centers for persistent psychiatric disorders, which provide an individualized therapeutic approach based on extra-hospital services.

**OBJECTIVES:**

We aimed to describe the clinico-epidemiological profiles of the patients seeking interventions at the CAPSIs.

**DESIGN AND SETTING:**

A cross-sectional study was conducted in two CAPSI in São Bernardo do Campo, SP, Brazil. One CAPSI is dedicated to the treatment of alcohol- and drug-related disorders, and the other to the treatment of other mental disorders.

**METHODS:**

In July 2017, we reviewed all active medical records of these two CAPSI, and collected the patients information including sex, race, education, type of referral, initial complaints, psychiatric diagnoses, and medication utilization.

**RESULTS:**

Of the 233 patients, 69.5% were male and 42.5% lived with their immediate family. Most of the patients were referred from other health services. Complaints on admission included agitation and aggressive behavior (30.9%). Autism spectrum disorder (ASD) was the most prevalent diagnosis (46.8%), followed by depressive disorder (13.8%). Of the patients, 81.5% were on regular medical follow-up and 70.3% were on a single medication only.

**CONCLUSION:**

Aggression complaints are the most prevalent in CAPSI, and diagnoses of ASD and psychotic disorders are more common. This situation differs from most CAPSI that present school complaints as the most prevalent, in which diagnoses of attention-deficit/hyperactivity disorder and conduct disorders are likely to be more frequent. The epidemiological profile of each CAPSI should guide the implementation of human and structural resources targeting the most prevalent complaints and diagnoses.

## INTRODUCTION

A child and adolescent mental health is one of the challenges of the Brazilian Psychiatric Reformation, and has become a public health issue involving the Brazilian Unified Health System (Sistema Único de Saúde, SUS).^1,2^

The Child and Adolescent Psychosocial Care Centers (Centro Estadual de Atenção Psicossocial e Infanto-Juvenil, CAPSIs) are dedicated to persistent psychiatric disorders and provide an individualized therapeutic approach based on extra-hospital services, such as therapeutic residences and outpatient clinic income-generation workshops, among others.^3,4^

In Brazil, the prevalence of mental health disorders among children and adolescents may vary from 7%–24.6%, with clinical intervention prevalence rates ranging from 4%–7.3%.^5,6^ However, limited studies have described the utilization of mental health services in this population.^7^

Lauridsen-Ribeiro et al.^8^ conducted a study on 141 children and adolescents (1–19 years old) and reported a 4.7% prevalence of mental disorders. Concomitantly, 74% of the affected patients did not seek for specific intervention before admission, 55.6% consulted a general hospital or service, and 12.9% consulted a psychologist.^8,9^

Mental illness among children and adolescents may be underestimated owing to a range of difficulties in discerning what is related to their development. Moreover, cultural and social factors may influence the qualitative and quantitative analysis of behaviors and symptoms.^10^ In addition, parents, teachers, and neighbors usually have distinct perceptions of the same problem, and the instruments employed for detecting early mental disorders may be poorly validated or standardized.^11^

Garcia et al.^11^ characterized the distribution and user profile of CAPSIs, and reported that 29.7% of the patients were diagnosed with behavioral disorders typical during childhood and adolescence, 23.6% with psychological development disorders, and 12.5% with intellectual disabilities. Moreover, 10.4% of the cases were neurotic, stress-related, and somatoform disorders and 1.8% of the patients have mental disorders due to psychoactive substances.^12^

São Bernardo do Campo (SBC) has approximately 850,000 inhabitants and hosts two CAPSIs, one dedicated to the treatment of alcohol and drug use disorders, and the other to mental health disorders.

The analysis of CAPSIs epidemiological data may suggest better strategies to improve CAPSI activities and promote tailored therapeutic interventions to reduce the impact of early disorders in the patient life course.

## OBJECTIVES

This study aimed to identify the prevalence of mental disorders of the patients in two CAPSIs in São Bernardo do Campo, Brazil, as well as describe their basic information such as sex, initial complaints, diagnoses according to the International Classification of Diseases (ICD-10), treatment adherence rates, and prescribed medications in the CAPSIs.

## METHODS

### Study setting

According to the Brazilian government, CAPSI is a facility for the assistance of children and adolescents who present with severe and persistent mental disorders, including substance use disorders. It serves cities and or regions with at least 70.000 inhabitants.^4^ Despite this definition, some large Brazilian cities, such as SBC, divide CAPSI into two different facilities for mental disorders and substance use disorders. These two services and the basic health units (BHU) are responsible for the entire city’s child and adolescent mental health care, with BHU managing mild mental disorders.

### Sample

SBC is a city in the metropolitan area of São Paulo, with an estimated population of 844.483 as of 2020.^13^ According to the last demographic census in 2010, 28.5% of the population aged 0–19 years.^13^

### Procedures

We performed a cross-sectional study in July 2017 by reviewing the medical records of the two CAPSIs. All patients treated by multidisciplinary staff and/or medical staff between January and July 2017 were included, and patients accessing a single evaluation were not included. A single evaluation usually means that they are evaluated and referred to another service.

The first, second, and third authors independently collected the data. Disagreements were resolved by group discussion until an inter-rater agreement of 100% was achieved. Missing data were further discussed with the professional of the CAPSI team, who was in charge of the patient.

An estimation of the prevalence of mental disorders and their correlates in this specific population has been provided.^14,15^

### Measures

Patient data from the medical records were obtained from a clinical protocol in place during the study period. The following information were collected for clinical reasons: Sex (male or female); race (white, black, Asian, native, or mixed); education (adequate for age, inadequate for age, school dropout, or school for special needs; type of referral (spontaneous or referred [e.g., basic health unit, school, rehabilitation centers, psychiatric emergency room, tutelary council, clinical emergency room units, private health services, technical guidance teams]); initial complaint (agitated/aggressive behavior, impairment in social interactions and neurodevelopmental delays, impairment in social interaction, neurodevelopmental delays, use of psychoactive substances, suicidal ideation, or suicide attempts; psychiatric diagnoses (autism spectrum disorder, acute psychotic disorder, depressive disorder, autism associated with intellectual disabilities, schizophrenia, intellectual disabilities, conduct disorder, attention-deficit/hyperactivity disorder, panic disorder, mental and behavioral disorders related to the use of multiple drugs, bipolar affective disorder, personality disorder, mental and behavioral disorders related to cannabis use, mental and behavioral disorders related to cocaine use, and obsessive-compulsive disorder); and medication utilization (antipsychotic monotherapy, antidepressants, psychostimulants, mood stabilizers, antihistamines, and benzodiazepines as monotherapy or in combination).

The computation and analyses of collected data were conducted using the Statistical Package for the Social Sciences (SPSS) version 15.0 (IBM Corp., Armonk, New York, United States).

### Ethics statement

Informed consent was obtained from both the patient and their legal guardians. This study was approved by the Faculdade de Medicina do ABC (FMABC) Ethics Committee (CAAE 2823.5719.3.0000.0082) dated June 19, 2020.

## RESULTS

We reviewed all active medical records of 233 patients (69.5% male and 30.5% female). The patient characteristics are shown in Table 1. Of the patients, 66.5% were white, 29.6% brown, and 2.6% black. Moreover, 43% of the patients are living with both parents, 46% with a single parent, 5.1% with other family members, 3.8% with shelters, and 2.1% with their grandparents.

**Table 1.** Epidemiological profile of the patients in CAPSIs in São Bernardo do Campo

Epidemiological profile			
Variable	n (233) % total		
**Sex**			
Male	162		**69.5**
Female	71		**30.5**
**Suitable school grade**	**Male**	**Female**	
Yes	93	40	**57.1**
No	69	31	**42.9**
**Family nucleus**			
Both parents	73	26	**42.5**
One parent	72	36	**46.4**
None	17	9	**11.1**
**Search for service**			
Spontaneous	46	15	**26.2**
Referred	116	56	**73.8**
**Medical follow-up**			
Regular	131	59	**81.5**
Irregular	31	12	**18.5**

CAPSI = Centros de Atenção Psicossocial (Child and Adolescent Psychosocial Care Centers).

Of the 233 patients, 57.1% were attending their expected school grade, 20.1% had failed at least one school year, 12.9% attended special schools, and 9.9% dropped out of school. School dropout was associated with specific ICD-10 diagnoses such as acute psychotic disorders (F23) and mental and behavioral disorders related to the use of multiple substances (F19), with a school dropout rate of 17.4%. In addition, the prevalence of drug addiction as a comorbidity among those who reported school dropout was 26.1%.

Diagnoses associated with school failures were as follows: depressive disorders (F32), 23.4% of cases; schizophrenia (F20), 12.8%; conduct disorders (F91), 8.5%; and conduct disorders associated with attention-deficit/hyperactivity disorder (F91 + F90), 8.5%. In addition, one case of attention-deficit/hyperactivity disorder (ADHD) with no associated comorbidity was the leading cause of school failure.

Notably, only a minority of patients sought medical help from the CAPSIs spontaneously, with 73.8% and 27.1% of them were referred from other services and BHU, respectively (Table 2).

**Table 2.** Referrals from other services to the CAPSI’s of São Bernardo do Campo

Referrals	Percent
Basic Health Units	27.1%
Not referred	24.9%
Others	12.1%
School	9.1%
Rehab Center	9.1%
Psychiatry Emergency Rooms	8.6%
Private Health Services	3.0%
Tutelary Council	2.2%
Clinical ER Units	2.0%
Technical Guidance Team	1.7%

CAPSI = Centros de Atenção Psicossocial (Child and Adolescent Psychosocial Care Centers).

Only 3.2% of patients reported disorders related to substance use. However, most were referred to the CAPSIs for other comorbidities, such as ADHD. We also observed that 4.2% of patients presented with substance use as an initial complaint but were not referred by the BHUs.

Common cause for referring to CAPSIs include agitated/aggressive behavior (30.9 %) and impairment in social interactions associated with neurodevelopmental delays (13.7 %) (Table 3). In the analysis of single symptoms as reasons for referral, we found that impairment in social interactions was the third most common cause of referral (12.4%), followed by neurodevelopmental delays (9.9%), chemical substance use (4.3%), suicidal ideation, suicide attempts (3.9%), and self-aggressiveness (3.4%). Other causes (21.5%) are presented in Table 3.

**Table 3.** Initial complaints of the patients in CAPSIs in São Bernardo do Campo

Initial complaints	Percent
Impairment in social interactions	12.4%
Neurodevelopmental delays	9.9%
Impairment in social interaction and neurodevelopmental delays	13.7%
Agitated/aggressive behavior	30.9%
Suicidal ideation or suicide attempts	3.9%
Self-aggressiveness	3.4%
Use of psychoactive substances	4.3%
Others	21.5%

CAPSI = Centros de Atenção Psicossocial (Child and Adolescent Psychosocial Care Centers).

ICD-10- based diagnoses include autism spectrum disorder (F84) (32.6%), depressive disorder (F32) (12%), and autism associated with intellectual disabilities (F84 + F79) (7.3%) (Table 4). Other diagnoses include schizophrenia (F20) (4.7%), intellectual disabilities (F79), conduct disorder (F91) (3.9%), ADHD (F90) (3%), panic disorder (F41) (2.6%), mental and behavioral disorders related to the use of multiple drugs (F19) (2.6%), bipolar affective disorder (F31) (2.1%), personality disorder (F60) (1.7%), acute psychotic disorder (F23) (1.7%), mental and behavioral disorders related to cannabis use (F12), mental and behavioral disorders related to cocaine use (F14), and obsessive-compulsive disorder (F42) (all 0.9%). The following are the rank of comorbidities: F90+ F91(3.9%), F84 + F62 (2.6%), F84+ F90 (2.6%), F19+ F91 (2.1%), F19 + F79 (1.3%). Several other comorbidities reported similar percentages: F20+ F79 (0.9%), F19+ F32 (0.9%), F79+ F90 (0.9%), F32+ F43 (0.9%), and F84+F79+G40 (0.9%). Meanwhile, 2.6% of the sample had unspecified diagnoses.

**Table 4.** Diagnoses of the patients in the CAPSIs in São Bernardo do Campo

Diagnoses	Percent
Autism Spectrum Disorder (F84)	32.6%
Autism Spectrum Disorder with Intellectual Disabilities (F84 + F79)	7.3%
Reactions to Severe Stress and Adaptation Disorders (F43)	0.4%
Panic Disorder (F41)	2.6%
Depressive Disorder (F32)	12.0%
Intellectual Disabilities (F79)	3.9%
Obsessive-Compulsive Disorder (F42)	0.9%
Mental and Behavioral Disorders Related to the Use of Multiple Drugs (F19)	2.6%
Attention-Deficit/Hyperactivity Disorder (F90)	3.0%
Conduct Disorder (F91)	3.9%
Attention-Deficit/Hyperactivity Disorder with Conduct Disorder (F90 + F91)	3.9%
Mental and Behavioral Disorders Related to Cannabis Use (F12)	0.9%
Autism Spectrum Disorder with Epilepsy (F84 + G40)	0.9%
Bipolar Affective Disorder (F31)	2.1%
Mental and Behavioral Disorders Related to the Use of Multiple Drugs with Intellectual Disabilities (F19 + F79)	1.3%
Autism Spectrum Disorder with Attention-Deficit/Hyperactivity Disorder (F84 + F90)	2.6%
Mental and Behavioral Disorders Related to the Use of Multiple Drugs with Conduct Disorder (F19 + F91)	1.7%
Schizophrenia (F20)	5.2%
Schizophrenia with Intellectual Disabilities (F20 + F79)	0.9%
Phobic-Anxious Disorders (F40)	1.7%
Acute and Transient Psychotic Disorders	1.7%
Others	2.6%
Autism Spectrum Disorder with Disorders of Habits and Impulses (F84 + F63)	2.1%
Autism Spectrum Disorder with Intellectual Disabilities with Attention-Deficit/Hyperactivity Disorder (F84 + F79 + F90)	0.9%
Attention-Deficit/Hyperactivity Disorder with Intellectual Disabilities (F90 + F79)	0.4%
Depressive Disorder with Reactions to Severe Stress and Adaptation Disorders (F32 + F43)	0.9%
Autism Spectrum Disorder with Intellectual Disabilities with Epilepsy (F84 + F79 + G40)	0.4%
Mental and Behavioral Disorders Related to the Use of Multiple Drugs with Depressive Disorder (F19 + F32)	0.9%

CAPSI = Centros de Atenção Psicossocial (Child and Adolescent Psychosocial Care Centers).

A total of 46.8% of patients were diagnosed with autism spectrum disorder (ASD), and 32.6% reported no associated comorbidities. Of these, 78.9% were male and 21.1% were female, with 31.2% aged 1–7 years. Among comorbidities related to ASD, 15% had intellectual disability, followed by ADHD (5.5%), and trichotillomania (4.5%). In general, 20.2% of the whole sample received multidisciplinary treatment without medical follow-up, whereas 65.1% of patients with ASD were taking psychotropic medications.

In addition, 81.5% of the sample was on regular medical follow-up (Table 1) and 70.3% on pharmacological treatment: antipsychotic monotherapy (25.8%), antidepressants (12%), psychostimulants (4.7%), mood stabilizers (2.2%), and antihistamines and benzodiazepines (0.4%). The following are the combination therapies: antidepressants and antipsychotics (9%), antipsychotics and mood stabilizers (5.6%), mood stabilizers + antidepressants (1.3%), psychostimulants + antipsychotics (1.3%), antipsychotics + benzodiazepines (0.9%), antidepressants and psychostimulants (0.4%), antidepressants + anticholinergic (0.4%), antidepressants + benzodiazepines (0.4%), and mood stabilizers + psychostimulants (0.4%). Combinations of the three medications were rated as 5.3% (Table 5 **)**.

**Table 5.** Main pharmacological therapies of the patients in CAPSIs in São Bernardo do Campo’s

Main pharmacological therapies	Percent
Antipsychotics	25.8%
Antidepressants	12.0%
Antipsychotics + Antidepressants	9.0%
Antipsychotics + Mood stabilizers	5.6%
Psychostimulants	4.7%
Mood stabilizers	2.2%
Others	40.7%

CAPSI = Centros de Atenção Psicossocial (Child and Adolescent Psychosocial Care Centers).

## DISCUSSION

We described the clinico-epidemiological profiles of the patients attending the two CAPSIs in SBC to better understand their characteristic, as limited literature has examined this specific population to date. In this study, patients attending CAPSIs in SBC were mostly male and have attended their expected school grade. This is consistent with a previous study,^16^ which reported a male rate of 66.1% based on an analysis of 248 medical records of patients in CAPSI in Rio de Janeiro state, Brazil.^16^ Aggressive/agitated behavior and ASD were the most prevalent initial complaints and diagnoses, respectively.

Most of the referrals to the CAPSIs were mainly BHUs, suggesting the active involvement of these units in the public health system. São Bernardo do Campo’s BHUs refer both externalizing and internalizing disorders to the CAPSI. Furthermore, most children and adolescents attended their expected school grade, indicating that patients could attend schools despite their mental health issues.

Our study found that psychomotor agitation was the main reason for referral. These externalizing behaviors are potentially urgent medical issues, which should be addressed by comprehensive child and adolescent mental health services, such as CAPSI.^17^ Delfinee & Reis, Beltrame & Boarini^18^, and Cunha^20^ found school issues to be the most prevalent initial complaints in their CAPSIs.^19^ We found that 4.3% of patients had psychoactive substance use as an initial complaint, whereas Hoffman et al.^3^ reported no substance abuse cases in their CAPSI sample. This may be due to São Bernardo do Campo’s city hosting a specific CAPSI to treat alcohol- and drug-related issues.

The prevalence of mental disorders found in our study remarkably differs from the findings of Hoffman et al.^3^ in Paraná state, Brazil. ASD was the most prevalent diagnosis in our sample, followed by depressive disorder. In contrast, Hoffman et al.^3^ reported behavioral and emotional disorders with onset usually occurring in childhood and adolescence (i.e., a broad ICD-10 category, which includes ADHD and conduct disorder) as the most prevalent (44.5%) in their CAPSI, followed by anxiety, dissociative, stress-related, somatoform, and other non-psychotic mental disorders (19.8%).

Medical follow-up of patients with CAPSI was regular in most cases. A predominance of antipsychotic medication use in monotherapy was found, which could be attributed to the aim of promoting patients’ adherence to treatments and reducing side effects.

This study was limited by a small sample size and short sampling period.

## CONCLUSION

The characteristics of the patients in the two CAPSIs in SBC had a different epidemiological profile from the CAPSI evaluated in previous studies. In CAPSI, such as in this study, in which complaints of aggression are the most prevalent, diagnoses of ASD and depressive disorders are more common. This situation differs from most CAPSI that present school complaints as the most prevalent, in which diagnoses of ADHD and conduct disorders are likely to be more frequent. Therefore, further studies of the clinico-epidemiological profile of CAPSIs are required. The epidemiological profile of each CAPSI should guide the implementation of human and structural resources targeting the most prevalent complaints and diagnoses.
